# Rate of diagnosed seasonal influenza in children with influenza-like illness: A cross-sectional study

**DOI:** 10.1371/journal.pone.0269804

**Published:** 2022-06-10

**Authors:** Mitsuo Uchida, Takenori Yamauchi

**Affiliations:** 1 Department of Public Health, Graduate School of Medicine, Gunma University, Maebashi, Gunma, Japan; 2 Department of Hygiene, Public Health and Preventive Medicine, Faculty of Medicine, Showa University, Tokyo, Japan; Kyoto University: Kyoto Daigaku, JAPAN

## Abstract

**Introduction:**

Although influenza surveillance systems have been used to monitor influenza epidemics, these systems generally evaluate diagnostic information obtained from medical institutions and they do not include patients who have not been examined. In contrast, community based epidemiological studies target people with influenza-like illness (ILI) that self-reported influenza-like symptoms whether they have medical examinations or not. Because the criteria for influenza surveillance systems and ILI differ, there is a gap between them. The purpose of this study was to clarify this gap using school-based survey data.

**Methods:**

Questionnaires about both ILI and the influenza diagnosis history during the 2018/19 season were administered to the guardians of 11,684 elementary schoolchildren in a single city in Japan. Based on their responses, a Bayesian model was constructed to estimate the probability of infection, ILI onset, and diagnosis at medical institutions.

**Results:**

Responses were obtained from guardians of 10,309 children (88.2%). Of these, 3,380 children (32.8%) had experienced ILI, with 2,380 (23.1%) diagnosed as influenza at a medical institution. Bayesian estimation showed that the probability of influenza cases being diagnosed among ILI symptomatic children was 70% (95% credible interval, 69–71%). Of the infected children, 5% were without ILI symptoms, with 11% of these patients diagnosed with influenza.

**Conclusions:**

This epidemiological study clarified the proportion gap between ILI and influenza diagnosis among schoolchildren. These results may help to establish epidemic control measures and secure sufficient medical resources.

## Introduction

Seasonal influenza is an upper respiratory tract infectious disease responsible for annual epidemics in children [1–3]. Surveillance systems for influenza have therefore been established in several countries to assess and control influenza epidemics [4–7]. These surveillance systems, which are based on the diagnosis of patients in medical institutions, have contributed to the detection of influenza virus subtypes and to the control of influenza epidemics.

Some people with influenza-like illness (ILI) do not seek medical care, therefore not all ILI affected individuals are included in influenza surveillance systems. Previous studies reported reasons why these individuals choose not to seek medical care include preference for over the counter (OTC) drug use [8], and background factors associated with season, geographical region, age group and gender [9]. Because identification of these patients is necessary to prevent the spread of influenza, community epidemiological surveys often include patients with ILI (e.g., ILINet [10] and Flu Near You [11]), in addition to those diagnosed in medical institutions. Epidemiological studies evaluating patients with ILI have also shown the impact of asymptomatic patients [12] and those with ILI [13] on the spread of infection, indicating that epidemiological surveys based on information from ILIs provide important insights for controlling epidemics. Because the criteria for ILI and for surveillance systems based on information from medical institutions differ, the interpretation of results can differ. Therefore, it is important to clarify the gap between ILI based on epidemiological surveys with the results of diagnosis in medical institutions.

Previous epidemiological studies in Japan have been performed to determine the proportion of elementary schoolchildren diagnosed with influenza in the community and the mode of epidemic spread [14], the impact of masks and vaccines on the prevention of disease [15], and the difference in hospitalization rates between vaccinated and unvaccinated children [16]. Despite being epidemiological surveys, those studies evaluated only children diagnosed at medical institutions, not children with ILI. Any gap between the rates of ILI and diagnosed influenza in children may have resulted in underestimations of influenza affects in previous studies. Other studies have studied differences between ILI and diagnosed cases, such as the effects of influenza diagnosis experience among ILINet participants [17], and the estimation of numbers of ILI cases based on influenza diagnosed cases using mathematical modeling [18]. However, there is sparse evidence available to evaluate the gap in the community making it difficult to determine any gap between the number of people with ILI and the number of influenza diagnosed cases.

The present study hypothesized that investigating rates of ILI and influenza diagnosis in schoolchildren would confirm the existence of a gap. Identification of this gap would allow for the quantitative determination of the relationship between these rates. This cross-sectional study of all elementary schoolchildren in one city in Japan during the 2018/2019 season compared the proportion of children who experienced ILI and the proportion of those who were diagnosed with influenza at medical institutions.

## Materials and methods

### Study subjects

To conduct a large-scale survey among schoolchildren, children attending all elementary schools in Isesaki City, Japan, were included. Isesaki City is located in Gunma Prefecture, in the Kanto Plain of central Japan, 60 meters above sea level, with a population of approximately 210,000 people and an area of 140 km^2^. The city is surrounded by other cities of similar size. There are no private elementary schools in the area, and 11,684 elementary schoolchildren attend 23 public elementary schools. A large municipal hospital is located in the center of the city, with clinics scattered throughout the area. In addition, each school has its own contracted school physician, and a system is in place to allow children with health problems to see the physician. Thus, there is no problem with access to medical care.

### Questionnaire

Questionnaires related to influenza were administered to guardians of all elementary schoolchildren via their homeroom teachers during the first week of March 2019. These questionnaires included questions about grade (1–6), sex (male/female), whether these children had experienced symptoms of ILI (temperature of 37.8°C or greater and cough and/or sore throat [19]) during this 2018/19 season (yes/no), and whether they had been diagnosed with seasonal influenza at a medical institution (yes/no). The deadline to submit the questionnaire was 1 week, with the questionnaires subsequently returned from teachers to the researcher. Families with more than one child were asked to complete a questionnaire for each child.

### Status of seasonal influenza

The National Institute of Infectious Diseases Japan reported that the timing of the influenza epidemic during the 2018/19 season was similar to that during previous years [20]. That, the epidemic spread around the week 50 of 2018, peaked around the week 4 of 2019, and almost ended around the end of February. The main subtype was AH1pdm09, followed by AH3, with type A being consistently prevalent nationwide. The official epidemic information of Gunma Prefecture reported that seasonal influenza in Isesaki City peaked during week 3 of 2019 [21]. In this study, the survey was conducted at the beginning of March, thus including almost all patients with ILI and influenza.

### Statistical analysis

All statistical analyses were performed using R software (ver. 4.0.5). The rates of ILI and of influenza diagnosed by medical institutions were compared using the Pearson’s Chi square test, with p < 0.05 defined as statistically significant.

**Fig 1:**
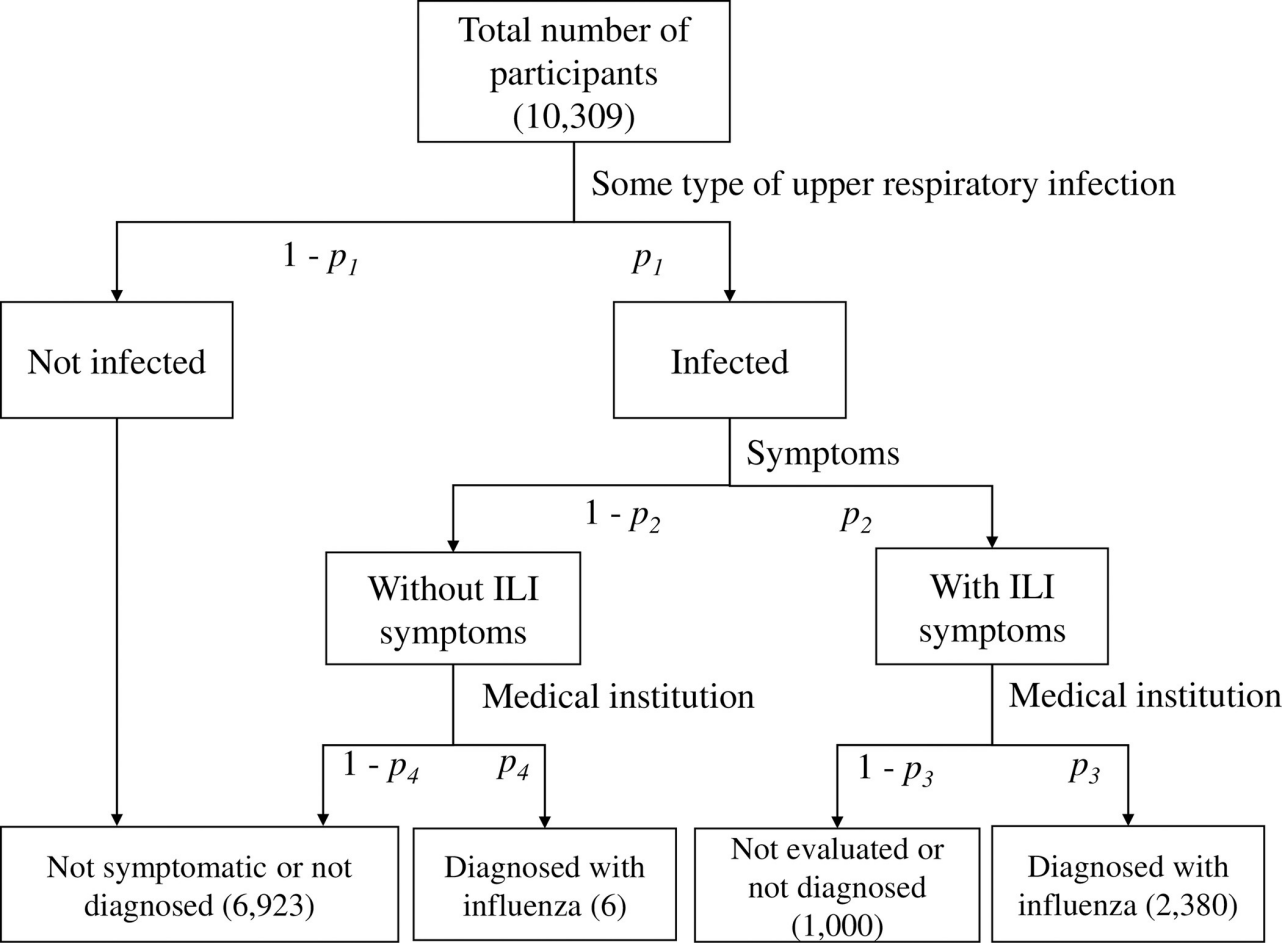
Flow chart of the study participants. All respondents were divided using three tiers: infection, ILI symptoms, and diagnosis of influenza. The probability of each was expressed as *p*_*1*_ to *p*_*4*_, and Bayesian estimation was performed.

The percentage of children with ILI who were diagnosed with influenza was determined and Bayesian analysis was performed. The probabilities of being infected and of not being infected with some type of upper respiratory tract infection were defined as *p*_*1*_ and 1-*p*_*1*_, respectively (Fig 1). Among infected patients, the probabilities of developing and not developing ILI were defined as *p*_*2*_ and 1-*p*_*2*_, respectively. The probabilities of patients with ILI being and not being diagnosed with seasonal influenza at a medical institution were defined as *p*_*3*_ and 1-*p*_*3*_, respectively. In addition, the probability of patients being diagnosed with influenza at a medical institution upon visiting a doctor for any reason other than ILI (e.g. headache, fatigue) was defined as *p*_*4*_, whereas their probability of not being diagnosed with influenza due to subclinical infection was defined as 1-*p*_*4*_.

Each probability in this flow was estimated with a Bayesian model using R software (ver. 4.0.5) and Rstan package (ver. 2.21.2). All probabilities were reported as point estimates and 95% credible intervals. We used uniform distribution for priors and the likelihood function was fitted to a Binomial distribution. Sampling was performed via four chains and each respective chain had 2,000 steps with 1,000 steps of burn in. Totally, random value was obtained from 4,000 steps data. Posterior distribution was estimated by the Hamiltonian Monte Carlo method, a type of Markov Chain Monte Carlo method. As a result of data sampling, all data were converged and all Rhats were below 1.1.

### Ethics approval

The study results were obtained anonymously, with no individual information obtained. Subjects who did not want to participate could choose not to respond to the questionnaire. The study procedure was reviewed and approved by the Committee for Medical Ethics of Gunma University (approval number 2018–087), which waived the requirement for informed consent.

## Results

Questionnaires were sent to the guardians of all 11,684 elementary schoolchildren, with the guardians of 10,309 (88.2%) children responding. Of the latter, 3,380 (32.8%) children had ILI, and 2,380 (23.1%) were diagnosed with influenza at a medical institution, whereas six without ILI were diagnosed with influenza (Table 1). Among diagnosed patients regarded as influenza cases, the sensitivity and specificity of ILI symptoms were 99.7% and 87.4%, respectively. ILI symptoms were significantly more frequent in boys than in girls and in children in younger than in older grades (Table 2). However, the proportion of those with ILI who were diagnosed did not differ significantly by grade or sex (Table 3). Therefore, grade and sex did not affect the proportion of diagnosed patients.

**Table 1:** A cross table of diagnosis of seasonal influenza and ILI symptoms among children.

	With ILI symptoms	Without ILI symptoms	Total
Diagnosed	2380	6	2386
Not diagnosed	1000	6923	7923
Total	3380	6929	10309

**Table 2:** Comparison of school grade and sex of children with and without ILI symptoms.

	With ILI symptoms	Without ILI symptoms	p-value#
Grade			
First	655	1004	p<0.001
Second	647	1057	
Third	595	1107	
Fourth	518	1182	
Fifth	513	1195	
Sixth	445	1329	
Sex			
Male	1732	3306	p = 0.006
Female	1633	3507	

# Chi-square test

Grade was undetermined in 62 children and sex was undetermined in 131 children, all of whom were excluded from the analysis.

**Table 3:** Comparison of school grade and sex of children with ILI symptoms by diagnosis of influenza.

	Diagnosed with influenza	Not diagnosed with influenza	Not responding	p-value#
Grade				
First	468	136	51	0.412
Second	466	119	62	
Third	411	133	51	
Fourth	358	106	54	
Fifth	374	94	45	
Sixth	299	106	40	
Sex				
Male	1224	340	168	0.164
Female	1150	350	133	

# Chi-square test

Grade was undetermined in seven children and sex was undetermined in 15 children, all of whom were excluded from the analysis.

The probability of each of the events in Fig 1 was estimated by Bayesian analysis. The values of *p*_*1*_ to *p*_*4*_ obtained by the analysis were 0.346 (95% credible interval [CI] 0.326–0.384), 0.950 (95% CI, 0.856–0.996), 0.702 (95% CI, 0.691–0.713), and 0.109 (95% CI, 0.009–0.443), respectively (Table 4). By conversion to proportions, these results indicated 70.2% of children with ILI symptoms were diagnosed with seasonal influenza at a medical institution. In addition, 5.0% of these children were without ILI symptoms, with 10.9% of these children being diagnosed with influenza.

**Table 4:** Bayesian model estimation.

	Mean	95% CI	SD
*p*_*1*_	0.346	[0.326–0.384]	0.017
*p*_*2*_	0.950	[0.856–0.996]	0.044
*p*_*3*_	0.702	[0.691–0.713]	0.005
*p*_*4*_	0.109	[0.009–0.443]	0.123

## Discussion

In this study, the history of ILI and diagnosis of influenza was obtained from a survey of all elementary schoolchildren in a mid-sized Japanese city. The proportion of children symptomatic for ILI who were diagnosed with seasonal influenza at a medical institution was estimated using a Bayesian model. The results showed that 70.2% of children with ILI were diagnosed with influenza at a medical institution. This study also evaluated the proportion of children without ILI symptoms and the proportion of the latter who were diagnosed with influenza. To our knowledge, this is the first study to focus on the gap between the rates of ILI and diagnosed influenza, and to estimate these rates among children in the community. This information may be useful in designing future influenza control measures and to estimate the potential number of influenza infected children in the community.

This census of elementary schoolchildren in a Japanese city found that 32.8% had ILI symptoms and 23.1% were diagnosed with influenza at medical institutions. Influenza surveillance in Japan includes sampling of patients at more than 400 medical institutions nationwide, allowing epidemic assessment in real time [22]. Over the past 10 years, the average number of reported patients per medical institution per week during the peak period of infection has ranged from 31.9 to 57.1 in Japan. These findings cannot be directly compared with the present results because of the lack of accurate information on the incidence of influenza in the community. The United States Centers for Disease Control and Prevention (CDC) estimated that the total numbers of patients with seasonal influenza over the past 10 years ranged from 9.3 to 45 million in the USA [23], but the wide range and the lack of age-specific information make these findings difficult to compare with the present results. The percentage of elementary schoolchildren in one Japanese City diagnosed with influenza at medical institutions during the 2014/15 season was reported to be 19.3% [14]. Although the rate of influenza diagnosis may vary by region and year, this rate among elementary schoolchildren was considered to be approximately 20%.

The results of the present study showed that 70% of elementary schoolchildren with ILI were diagnosed with influenza, indicating a 30% gap between ILI symptoms and influenza diagnosis. Conversely, the number of people with ILI in the community was about 1.4 times greater than the number of people diagnosed at a medical institution. A study estimated whole ILI cases using H1N1pdm diagnosed serum samples and reported medical attendance rates of 90.9% in children aged 5 to 9 years old and 79.7% in those aged 10 to 14 years old among ILI cases [18]. Although the numbers in the present questionnaire survey study were lower than those in the previous study, both studies report a 10% to 30% discrepancy between ILI and diagnosed cases in school-aged children.

This discrepancy between the proportion of children with ILI and the proportion of diagnosed children may be caused by individual behavior or individual background factors. Reasons for not seeking medical care even when ILI was present have been reported in previous studies, including OTC drug use [8], and background factors such as season, geographical region, age group, and gender [9]. Factors regarding choice of behavior affecting disease prevention may include family size [24], symptoms such as fever and rash [25] and malaise [26]. In addition, the decision to seek medical care might be influenced by perception of the surrounding epidemic situation of influenza or anticipation of effects of infection control measures such as school closures. For example, a previous study reported that school closures due to influenza affected attitudes and behaviors of children’s guardians [27]. Because infection control measures such school closures may require guardians to take leave from work without their schedule, they may pay attention to the influenza epidemic and seek medical care more than usual to avoid those unscheduled leave. Another factor affecting the gap between the proportion of people with ILI and the proportion of diagnosed influenza cases may include diagnostic technology, wherein the sensitivity of immunochromatography diagnosis kits usually used for rapid influenza diagnosis in Japan is around 70% [28] to 80% [29]. In addition, ILI is also caused by other upper respiratory infections such as rhinoviruses, enteroviruses, and adenoviruses [30], which may explain the discrepancy between ILI and influenza diagnosis rates. Some of the other upper respiratory infection data were recorded in official surveillance data [21], and a check of 2018/19 season data revealed the numbers of other upper respiratory infection reports were sufficiently relatively small to conclude they had little or no impact on the current study result. Thus, factors affecting the gap between ILI symptoms and influenza diagnosis rates may include both individual differences in background factors and medical diagnostic methods. Although the present study did not directly evaluate children’s behavior or other diagnostic results, it showed a disparity in the proportion of children with ILI and those diagnosed with influenza.

A few patients in the present study were diagnosed with influenza in the absence of ILI. The proportion of subclinical influenza infections has been reported to range from 0–20% [31], with another study reporting that about 50% of patients with influenza were asymptomatic [32]. To detect all infected people, including those with subclinical infection, it is necessary to test the entire population for antibodies, a procedure that is generally difficult. This study estimated the proportion of asymptomatic patients using a mathematical approach, finding that 5% of infected people become asymptomatic without developing the disease. Moreover, 11% of asymptomatic children were diagnosed with influenza. Although these data have a limitation in that the estimation was derived using a simulation model based on a small number of cases, the results are important to consider the risk of influenza cases without ILI symptoms. Those patients had been unaware that they had influenza, but went to a doctor due to other physical conditions, such as a headache or abdominal pain, which may have led to the incidental detection of influenza. There may be not an insignificantly small number of asymptomatic patients, and we have to consider how to include them in the surveillance system to control influenza epidemics.

We determined ILI cases by yes/no responses in this study, therefore participants who reported ILI at least once in 2018/19 season were regarded as cases. However, because study participants might experience multiple ILI in a season, we add a caution to our interpretation of the study result. For example, other reports such as ILINet in USA [10], count total ILI visits as case numbers in a medical institution. Thus, if we compare the current study results with numbers of total ILI visit numbers such ILINet, our result may underestimate ILI frequency. However, the aim of this study is not to know the frequency of ILI but to know the gap between the proportion of people who experienced ILI and the proportion of people diagnosed with influenza in a certain population. Because we focused on the participants who experienced ILI at least once and defined them as ILI cases for comparison, there should be no significant impact on interpretation of the study results regarding whether participants experience multiple ILI or not. Furthermore, to analyze ILI experience case numbers than total multiple ILI counts is better to show accurate comparison using same dimension or unit of influenza diagnosed case numbers. Thus, the definition of ILI cases in this study is thought to be valid to clarify the current study aim. If the frequency of ILI is analyzed in a future study, we will ask about multiple ILI experience in the study subject.

This study had several other limitations. First, subjects who did not go to a medical institution may not have responded to the questionnaire, resulting in an underestimation of patients diagnosed with influenza. Furthermore, the accuracy of the responses may have been reduced due to recall bias. Second, the present study did not include serological diagnoses of influenza. Rather, patients diagnosed in medical institutions were regarded as having influenza. Therefore, the proportion of asymptomatic patients may have been lower than in studies based on serological tests. In addition, the method of influenza diagnosis at the medical institution was not assessed, preventing evaluation of whether infections were diagnosed by rapid kits or by clinical symptoms. Third, this study could not distinguish between subjects who did not seek medical attention after the onset of ILI and subjects who were not diagnosed with influenza at medical institutions despite having ILI symptoms. That is, the 1,000 subjects who were not diagnosed with influenza included both these two groups of subjects. To understand these behaviors, we need to add a question in a further study to determine whether ILI symptomatic children attended a medical institution or not.

## Conclusion

This present study estimated the probability of influenza diagnosis based on the proportion of children with ILI. We found a difference between the proportion of children that report ILI symptoms and the proportion of children diagnosed with influenza. This may explain the gap between influenza surveillance system and epidemiological studies based on ILI. The result of this study provides important information wherein the gap could be used as an indication for taking countermeasures such as preparing medical resources for potential ILI patients against infectious disease epidemics in the community.
